# Decentralized facility financing versus performance-based payments in primary health care: a large-scale randomized controlled trial in Nigeria

**DOI:** 10.1186/s12916-021-02092-4

**Published:** 2021-09-21

**Authors:** Madhulika Khanna, Benjamin Loevinsohn, Elina Pradhan, Opeyemi Fadeyibi, Kevin McGee, Oluwole Odutolu, Gyorgy Bela Fritsche, Emmanuel Meribole, Christel M. J. Vermeersch, Eeshani Kandpal

**Affiliations:** 1 The MacMillan Center, Yale University, New Haven, USA; 2 The Global Fund, Global Health Campus, Chemin du Pommier 40, 1218 Grand-Saconnex, Geneva, Switzerland; 3 Health, Nutrition and Population, The World Bank, 1818 H Street NW, Washington, DC 20433 USA; 4 Health, Nutrition and Population, The World Bank, 102 Yakubu Gowon Cres, Asokoro, Abuja, Nigeria; 5 Development Data Group, The World Bank, 1818 H Street NW, Washington, DC 20433 USA; 6 The Federal Ministry of Health of Nigeria, New Federal Secretariat Complex, Phase III, Ahmadu Bello Way, Central Business District, FCT, Abuja, Nigeria; 7 Development Research Group, The World Bank, 1818 H Street NW, Washington, DC 20433 USA

**Keywords:** Health financing, Maternal and child health, Quality of care, Nigeria

## Abstract

**Background:**

Health system financing presents a challenge in many developing countries. We assessed two reform packages, performance-based financing (PBF) and direct facility financing (DFF), against each other and business-as-usual for maternal and child healthcare (MCH) provision in Nigeria.

**Methods:**

We sampled 571 facilities (269 in PBF; 302 in DFF) in 52 districts randomly assigned to PBF or DFF, and 215 facilities in 25 observable-matched control districts. PBF facilities received $2 ($1 for operating grants plus $1 for bonuses) for every $1 received by DFF facilities (operating grants alone). Both received autonomy, supervision, and enhanced community engagement, isolating the impact of additional performance-linked facility and health worker payments. Facilities and households with recent pregnancies in facility catchments were surveyed at baseline (2014) and endline (2017). Outcomes were Penta3 immunization, institutional deliveries, modern contraceptive prevalence rate (mCPR), four-plus antenatal care (ANC) visits, insecticide-treated mosquito net (ITN) use by under-fives, and directly observed quality of care (QOC). We estimated difference-in-differences with state fixed effects and clustered standard errors.

**Results:**

PBF increased institutional deliveries by 10% points over DFF and 7% over business-as-usual (*p*<0.01). PBF and DFF were more effective than business-as-usual for Penta3 (*p*<0.05 and *p*<0.01, respectively); PBF also for mCPR (*p*<0.05). Twenty-one of 26 QOC indicators improved in both PBF and DFF relative to business-as-usual (*p*<0.05). However, except for deliveries, PBF was as or less effective than DFF: Penta3 immunization and ITN use were each 6% less than DFF (*p*<0.1 for both) and QOC gains were also comparable. Utilization gains come from the middle of the rural wealth distribution (*p*<0.05).

**Conclusions:**

Our findings show that both PBF and DFF represent significant improvements over business-as-usual for service provision and quality of care. However, except for institutional delivery, PBF and DFF do not differ from each other despite PBF disbursing $2 for every dollar disbursed by DFF. These findings highlight the importance of direct facility financing and decentralization in improving PHC and suggest potential complementarities between the two approaches in strengthening MCH service delivery.

**Trial registration:**

ClinicalTrials.gov NCT03890653; May 8, 2017. Retrospectively registered.

**Supplementary Information:**

The online version contains supplementary material available at 10.1186/s12916-021-02092-4.

## Background

The sustainable and equitable financing of high-quality health systems remains a central challenge towards achieving universal health coverage (UHC) [1–7]. Further, low health worker performance and motivation are key contributors to poor healthcare in developing countries [8, 9]. Tying payments of health facilities to performance on predefined indicators in the form of performance-based financing (PBF) has been hypothesized as a possible strategy to improve maternal and child health (MCH) in low-income countries, including Burundi, Rwanda, Argentina, and Zimbabwe [10–19]. These PBF interventions are typically part of a broader health system reform—that includes autonomy, supervision, and monitoring [20, 21]. Thus, based on the early evidence from Rwanda and the persistent conundrum of health system financing, many donors and lending agencies encouraged national governments to adopt PBF in an attempt to improve efficiency and the quality of care [22, 23]. Indeed, 35 PBF pilots were funded by the World Bank’s Health Results Innovation Trust Fund, largely in sub-Saharan African countries, 29 of which were accompanied by impact evaluations [24, 25]. However, mixed evidence of effectiveness from these impact evaluations highlights unevenness in the impact of PBF programs in improving MCH coverage or quality [25, 26]. There may be many reasons for this unevenness: as a broad-based health system reform, PBF is a complex intervention. Indeed, questions have been raised about the ease of implementation of PBF relative to decentralized financing approaches, its impacts on equity, the heterogeneity in payment schemes and program design, and impacts on worker motivation, as well as the system-wide and long-term impacts of PBF programs and whether short-run interventions that lack stakeholder ownership can damage health systems [27–31]. Results also highlight the importance of other approaches that include user-fee removal or even just community oversight—whether in combination with supply-side financial incentives or not [32, 33].

Further, when comparing PBF with direct facility financing (DFF) that disbursed as much as PBF but had lower administrative costs, evidence from Cameroon suggests that different models of DFF may be as effective as PBF at increasing health service coverage [34]. In addition, evidence from Zambia suggests that DFF may be more cost-effective than PBF because, by not requiring direct measurement of outputs or third-party verification visits to facilities, DFF has lower administrative costs [35]. DFF also has the benefit of causing fewer unintended effects, for instance by not incentivizing gaming [36].

This study reports on the results of a trial in Nigeria to shed light on two important questions related to health system financing in developing countries. The first is whether two popular approaches—PBF and DFF—are superior to business-as-usual. The second is how PBF and DFF compare with each other in terms of impact on MCH service provision. In this context, the DFF intervention implemented was akin to the PBF intervention and thus a health system reform of its own. The DFF arm in Nigeria, unlike those in Cameroon and Zambia, incorporates identical accountability, autonomy, community engagement, and supervision elements as the PBF intervention. In addition, both PBF and DFF provided equal decentralized operating grants. However, only PBF provided an additional incentive-based payment to health workers based on the quantity and quality of the services they provided, which was equal in amount to the decentralized operating grants. Thus, unlike the previous studies, PBF gave health facilities $2 for every $1 disbursed by DFF. This design feature allows us to cleanly estimate the impact of performance-based financial incentives and additional funding over and above an otherwise identical decentralized financing alternative. PBF and DFF transferred funds directly to the bank accounts of individual publicly owned health facilities and gave facilities substantial autonomy in how they used the money, engaged community leaders in facility management, and strengthened supervision by increasing its frequency and introducing a quantified supervisory checklist (QSC).

The decentralization setup and Public Finance Management (PFM) legal environment also distinguish the intervention studied here from the literature. For instance, the public budget is much more centralized in Cameroon and Zambia than in Nigeria [34, 35]. Further, the literature from other heavily devolved African countries like South Africa and Kenya suggests that there is a likelihood of recentralization at the sub-national level, with decreased autonomy at service delivery points [30]. This in turn results in an even stronger need for facility-level managerial autonomy. However, some PFM arrangements in Nigeria, including a sub-national application of PFM rules around Treasury Single Accounts may mean that resources from incentives may not always reach the intended beneficiary. While this study cannot disentangle the effects of the accompanying components of enhanced autonomy, supervision, and community engagement from the changes in financing to the PBF and DFF arms, these components differentiate the Nigerian trial from those previously studied in the literature.

There are several reasons why this study is important: (i) it documents the role of paying-for-performance and the potential complementarities in two popular health financing approaches, PBF and DFF, when both treatment arms are accompanied by autonomy, community engagement, and enhanced supervision; (ii) it likely does not suffer from pilot test bias because it was implemented on a large scale (covering over 9 million people) for a fairly long period (3 years) and at an incremental cost that is likely affordable using domestic resources; and (iii) it provides important design elements for the Basic Health Care Provision Fund under the National Health Act, which attracted US$180 million of additional domestic resources for primary health care (PHC) in the 2018–2019 federal budget.

The context is also important: Nigeria has made only modest progress on health outcomes in the last decade and ranks 152nd out of 157th on the World Bank’s Human Capital Index [37]. A poor child in Nigeria faces the highest risk of dying before his or her fifth birthday in all of West Africa; the country will soon overtake India as having the largest number of under-five deaths in the world despite having fewer children and a much smaller population (191 million compared with 1.34 billion) [38, 39]. The maternal mortality ratio remains at 576 deaths per 100,000 live births and represents 12.5% of the global maternal mortality burden [40, 41]. This sluggish progress on health outcomes is consistent with slow progress on MCH service delivery. In 1990, a Demographic and Health Survey (DHS) found immunization coverage (using the third dose of the Diphtheria, Pertussis, and Tetanus vaccines as a proxy for complete immunization) to be 33% [42]. It was also 33% in 2016 [43]. Similarly, slow progress has been noted for antenatal care (ANC), skilled birth attendance, and the contraceptive prevalence rate (CPR). In such contexts, DFF-type interventions that are relatively easy to implement and cost less than PBF may hold a lot of appeals.

## Methods

### Approaches studied

This study comprised two treatment arms: PBF and DFF. First, we describe the PBF intervention. In the PBF arm, facilities received a quarterly payment based on the quantity of predefined MCH services they provided. Each type of service had a tariff associated with it, and the facility received a payment that reflected the number of services provided multiplied by the tariff. For example, if a PHC facility fully immunized 100 children in the quarter and the tariff was 500 Nigerian naira per child immunized, the facility would receive 50,000 Nigerian naira. The quantity of services was reported and verified monthly by an external verification agency. To address quality of care (QOC), a QSC measuring facility quality was used by district supervisors on a quarterly basis. The visit to implement the QSC formed the core of the enhanced supervision received by PBF and DFF [44, 45]. PBF and DFF also received quarterly training tied to the QSC. Specifically, during the QSC visit, the supervisors first assessed the QSC at the facility and then fed the results back to health workers during the same visit. Next, using the results of the QSC, the supervisors engaged in problem-solving with health facility staff as well as discussed and trained facility staff on the key dimensions of facility quality in the QSC. Finally, facility managers were provided training every 6 months on best practices of facility management and financial administration.

The QSC assessed structural and process QOC and formed the basis of a quality bonus. Facilities could receive up to a 25% quality bonus scaled by the improvement over their previous quarter’s performance. This bonus was calculated over the earnings based on quantity alone. If the quality score was 100%, the facility would earn an additional 25% of the volume earnings over the past quarter. If quality were 50%, then the quality bonus would be an additional 12.5% (25%*50%) of the volume earnings. There was no upper limit for individual health facilities; the actuarial model used an annual global budget for the entire project area. The QSC was also separately verified by an external verification agency. Since the QSC visit and verification were not on the same day, verification reports within 10% of the quantities reported in the QSC were tolerated. Any reports that were more than 10% apart for more than 3 months in a row resulted in an audit of the facility by the Federal Ministry of Health. (The checklist and quarterly scores received by each of the 1389 facilities and the associated payment are published online [46].) In the PBF arm, an additional bonus was tied to the facility’s remoteness; this bonus could be up to 40% depending on the distance from the local governance area’s (LGA) administrative center [47]. The payment formula annex (Additional file 1: Table 1) provides an example of how the payment was calculated.

The amount earned by the facility was transferred electronically to the facility’s bank account for which the signatories were the officer in charge of the facility (typically a nurse or public health officer) and the chair of the Ward Development Committee (WDC). The WDC is a pre-existing community group that addresses development challenges for a population of 10,000–20,000 people. Facilities could use the funds they earned for (i) health facility operating costs (at least 50%), including maintenance and repair, drugs, and consumables (which they could purchase only from pre-qualified pharmacies), outreach, and other quality enhancement measures; and (ii) performance bonuses for health workers (up to 50% of the amount earned). The performance bonuses added about 10–20% to the health worker’s salaries.

Next, we describe the DFF arm. DFF was identical to PBF except that the payments to the PHC facilities were not linked to the quantity or quality of services they delivered, and no performance bonuses were paid to the health workers (Table 1). The amount of funds transferred to the DFF facilities was set, by design, to be exactly half of the average of what PBF facilities in the same state earned. This was done retrospectively: DFF income was entirely dependent on the actual disbursements to PBF facilities over the past quarter. The PBF prices were set prospectively, but there was no cap on volumes at the facility level.

**Table 1:** Summary of program: Treatment arms

Characteristic	PBF	DFF	Control
Funds electronically transferred to a health facility account	Yes	Yes	No
Autonomy of facility to allocate funds	Yes	Yes	No
Community engagement in facility management	Yes	Yes	No
Enhanced supervision using a quantitative supervisory checklist	Yes	Yes	No
Facility payment linked to quantity and quality of services	Yes	No	No
Remoteness bonus	Yes	No	No
Salary bonuses to health workers based on performance	Yes	No	No
Level of overall incremental funding (US$ per capita per year)	$3.49	$1.74	$0.00

Summary of the program features of the three arms of the program—performance-based financing, direct facility financing, and business-as-usual or control

DFF facilities were subject to the same reporting requirements as PBF facilities but not to the monthly third-party verification of quantity or quality. However, the DFF facilities had the same level of autonomy in using their funds as PBF, had to have WDC participate in the facility management committee, were supervised in a similar way, and also received funds in their bank accounts through electronic transfer. The program was implemented by the National Primary Health Care Development Agency at the federal level, state primary health care development agencies (SPHCDAs) at the state level, and PHC departments at the district level (called LGA in Nigeria) [48].

In both PBF and DFF, income from all sources—including user fees and PBF or DFF disbursements—was merged in one bank account and managed together. Conceptually, PBF and DFF were leveraging mechanisms for input financing and user fees. In PBF facilities, providers were actively encouraged to lower user fees as a strategy to boost demand and in some cases waive out-of-pocket fees.

### Study design

**Fig. 1:**
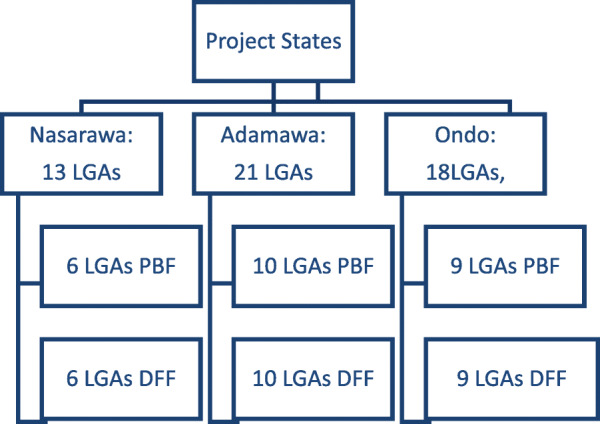
Randomization. The flow of participating districts (Local Governance Authorities or LGAs) in the study

As noted in the study protocol, which is included in the supplementary materials, the intervention states were chosen based on (1) greater health needs, (2) states with relatively high implementation capacity, (3) willingness to implement performance financing approaches, and (4) geo-political representation and filling gaps in donor support (as well as avoiding duplication). Thus, the results may be indicative of our treatment sample and not the entire country. However, within the states, the study randomly allocated all 52 districts (comprising about 250,000 population, on average, for a total treated population of over 9 million) in the experimental states (Adamawa, Nasarawa, and Ondo) to either the PBF or DFF arms. Thus, the comparison of PBF and DFF groups constitutes a cluster randomized trial. Figure 1 below describes the randomization process that assigned districts or LGAs in the three treatment states to one of these arms. The program was implemented in 1389 facilities (PBF arm=709; DFF arm=680) in all the districts in Adamawa, Nasarawa, and Ondo. The treated districts were randomly assigned with equal probability, by computer, to PBF or DFF. All primary health facilities in the treated districts received the assigned intervention.

For the evaluation, a population proportional subset of treated facilities was sampled in each of the treated LGAs. In Nasarawa, this yielded a sample of 72 facilities each in PBF and DFF; 97 PBF and 96 DFF facilities in Ondo, and 107 PBF and 106 DFF facilities in Adamawa. In addition, the impact evaluation established a control group (business-as-usual) by selecting states resembling the experimental states along 13 observable demographic characteristics that are associated with PHC outcomes. Further details are provided in the balance annex (Table 1-3; Additional file 2). The three control states were neighbors of the experimental states in the same geopolitical zone: Taraba (North East), Benue (North Central), and Ogun (South West). Within these states, LGAs were randomly selected to be sampled in the business-as-usual arm. This process yielded 8 LGAs and 107 facilities in Taraba, 7 LGAs and 72 facilities in Benue, and 10 LGAs and 97 facilities in Ogun. The comparison of PBF and DFF versus the control states was thus a “difference-in-differences” assessment.

Household surveys and health facility surveys were undertaken in all districts of the three study states both at baseline and endline. Baseline data were collected between February and April of 2014; the endline data were collected between August and October of 2017. Since the rollout of PBF and DFF occurred in July 2014, the study assesses 3 years of implementation. Choosing districts as the unit of treatment reflects the way funds are disbursed in the Nigerian health system but also aids in minimizing contamination from health facilities in one treatment arm to those in another. Districts are large—they are divided into between 10 and 20 wards and have an average population of between 150,000 and 250,000 inhabitants, which mitigates the risk of contamination.

### Study sample

For the study, health facility surveys were conducted by the National Bureau of Statistics in one randomly selected health facility per ward (there are 10–15 wards per district), resulting in baseline and endline surveys of 786 health facilities (41% of all treated facilities) across the two experimental arms. As summarized in Table 2, these surveys included (i) an assessment of the health facility itself (availability of drugs, equipment, etc.), (ii) an interview with at least one health care provider at the facility (not necessarily the same health worker at baseline and endline), (iii) direct observation of patient-provider interactions in the context of ANC and under-five curative care visits, and (iv) exit interviews with ANC and under-five patients.

**Table 2:** Sample characteristics

Characteristic	PBF	DFF	Control
Number of districts	23	25	25
**Baseline**			
Number of health facilities implementing the approach	709	680	NA
Number of health facilities surveyed	269	302	215
Number of providers interviewed	804	827	619
Number of ANC patients observed and interviewed	727	784	445
Number of under-five curative care patients observed and interviewed	735	670	373
Number of households surveyed	2538	2748	2804
**Endline**			
Number of health facilities surveyed	269	302	215
Number of providers interviewed	981	855	521
Number of ANC patients observed and interviewed	874	1077	594
Number of under-five curative care patients observed and interviewed	928	1138	509
Number of households surveyed	2514	2677	2816
Number of patients observed			

Description of the study sample: numbers of districts, health facilities, providers, ANC and curative care patients, and households surveyed, and patients observed through direct clinical observation

The study protocol, presented in online supplementary materials, notes that this sampling design was powered to detect a treatment effect of 10 percentage points with 97% certainty for facility-level outcomes and with 90% certainty for household-level characteristics. Indeed, the standard errors reported here, clustered at the district level, suggest that the study is reasonably well powered.

The National Population Commission of Nigeria defined enumeration areas across the country for the 2006 census. In 2008, the Federal Ministry of Health used these enumeration areas to create facility catchment areas. For this study, all households in the catchment areas of the sampled facilities were listed at baseline in 2014 and again at endline in 2017. From the full household listing, we sampled all households with at least one pregnant woman or a birth in the past 24 months on a probability-proportional-to-size basis. Note that if patients respond to perceived health facility quality improvements from either PBF or DFF by traveling further to attend a more distant health facility, then we might be underestimating the impact of the interventions.

The total sample sizes were 8090 households at baseline and 8007 at endline. The household questionnaire collected data on socioeconomic and demographic conditions along with detailed health histories including the woman’s pregnancy and delivery.

### Outcomes

The study protocol (Table 5 of the protocol provided in the supplementary material) identified five quantitative indicators as primary outcomes: (i) modern CPR, (ii) ANC, (iii) institutional delivery, (iv) Penta3 immunization of children 12–23 months old, and (v) use of insecticide-treated mosquito nets (ITNs) by under-fives. In addition, the protocol (Table 6 of the protocol) listed a series of 26 specific measures of QOC that included structural measures (e.g., availability of essential drugs and contraceptives) and process measures (such as health worker knowledge and the extent to which national protocols were followed).

### Statistical analysis

To estimate the impact of PBF versus DFF, this study uses a difference-in-differences approach within an experimental cluster randomized controlled trial (hereafter, experimental DiD). The DiD approach compares the changes from baseline to endline in one project arm to the same change in the other arm. We also included state fixed effects in all specifications and facility fixed effects where appropriate. Standard errors were clustered at the district level for facility-level data and at the enumeration area level for household-level data. Observations missing an observation for either baseline or endline for any of the outcomes examined are excluded from the analysis. The estimation equation used is as follows:

$$ {y}_{ijst}={\beta}_0+{\beta}_1\ast {Treatment}_{js}+\kern0.5em {\beta}_2\ast {Post}_t+\kern0.5em {\beta}_3\ast {Treatment}_{js}\ast {Post}_t+{\delta}_s+\kern0.5em {\varepsilon}_{ijst} $$

While the PBF and DFF comparison relies on a randomized control trial, the business-as-usual districts were chosen using matching on observables. The DiD results for the control comparison are thus quasi-experimental. Thus, we tested the parallel trend assumption underlying DiD using data from the 2008 and 2013 Demographic and Health Survey. As shown in online supplementary materials, the PBF and DFF arms are generally balanced. However, as also shown in online supplementary materials, in the comparison of the control arm to the treatment arms, 8 of 22 key household characteristics were significantly different. Crucially, these 8 unbalanced indicators include skilled birth attendance (SBA). Thus, we adjusted the DiD estimates comparing treatment to business-as-usual to match treatment and control units on baseline characteristics to estimate the probability that a unit falls into either a project or a control state [26]. This probability was then used to reweight the DiD regression equation by placing a greater weight on the treated observations that look more like the control observations. However, it is worth noting that in only 1 out of 22 comparisons control states are worse off than experimental states, suggesting that, if anything, unadjusted DiD underestimates the true impact of PBF and DFF interventions. Further details are provided in the parallel trends and balance annex (Tables 1-3; Additional file 3).

Finally, we use a wealth index to assess the equity implications of PBF versus DFF. For comparability to a nationally representative sample, the wealth index recreates the asset index from the 2013 DHS. A principal components analysis on DHS data yielded individual asset weights that were then applied to our survey data to create a wealth index, which was then divided into five quintiles at the same cutoffs as the DHS data.

## Results

Participants were recruited between January 15, 2014, and May 31, 2014, at baseline, and July 20, 2017, and October 31, 2017, at endline. Table 3 describes the changes in the quantitative indicators highlighted in the study protocol. Compared with control, the quantity-related indicators identified in the protocol showed positive adjusted DiDs in six out of ten cases; four of these six were statistically significant at *p*<0.10. Given that there were a few differences between the treatment and control arms at baseline, in alternative specifications, we controlled for baseline levels of the considered indicators; results are robust. Leveraging the randomized assignment to PBF or DFF, the results show that PBF outperformed DFF on institutional delivery, and the share of institutional deliveries in PBF areas was 10% higher. On the other hand, DFF did better on Penta3 immunization and ITN use by under-fives. Detailed results are provided in Table 1 of Additional file 3. The facility equivalents of these indicators are reported in Table 2a of Additional file 3, while detailed results of impact on skilled birth attendance and institutional delivery are reported in Table 2b of Additional file 3.

**Table 3:** Impact on utilization indicators

Indicator	Baseline coverage			Change from baseline			Quasi-experimental adjusted DiD		Experimental DiD
	Control	PBF	DFF	Control	PBF	DFF	PBF vs. control	DFF vs. control	PBF vs. DFF
mCPR (%)	18.9	17.7	16.2	2.2	9.8	7.6	5.7**	3.3	2.1
Penta3 immunization (%)	35.9	47.9	33.1	−0.3	11.9	19.3	11.1**	20.6***	−6.0*
Institutional delivery (%)	55.8	47.7	54.4	4.2	17.9	7.7	6.7*	−3.1	10.1***
At least 4 ANC visits (%)	46.0	51.9	52.1	4.1	2.8	5.1	−3.7	−3.5	−2.5
ITN use by under-fives (%)	45.6	46.6	42.5	18.9	15.7	19.7	−0.9	3.2	−5.6*
Out of pocket payment for curative care on children under-five (in naira)	261.8	222.5	201.9	−14.45	110.8	233.9	59.6	237.1	−122.2
Winsorized out of pocket payment for curative care on children under-five (in naira)	58.85	222.5	201.9	188.53	110.8	233.9	7.2	17.9**	−11.7

The impacts of the program on service utilization for antenatal care and curative care for children younger than 5 years of age. ^*^p<0.1, ^**^p<0.05, and ^***^p<0.01. Due to the presence of influential outliers, we also present the out-of-pocket payment winsorized at 5%

Table 4 shows the effects of PBF and DFF on the examined indicators broken down by wealth quintiles. Many large improvements in service utilization observed in the PBF or DFF arms came from impacts on women in the third, fourth, and fifth wealth quintiles. Relatively, little impact is observed in the two poorest quintiles of wealth. Note, however, that over 85% of the Nigerian population is among “the global bottom 40%,” so even the highest wealth quintile in this environment represents some of the world’s poorest people [41]. (Detailed results are provided in Tables 3 and 4 of Additional file 3) Compared to DFF, PBF increases institutional delivery by 22 percentage points (pps) in the second wealth quintile and 9 pps in the third, and 14 pps in the fourth, but had no additional impact on the poorest quintile. Similarly, compared to PBF, DFF improved ITN usage for those in the top three wealth quintiles. Though imprecise, relative improvement in DFF was the largest in the poorest quintile but was also substantial in the top three wealth quintiles.

**Table 4:** Impact on utilization indicators by wealth quintile

Indicator	Group	Q1 (poorest)	Q2	Q3	Q4	Q5 (richest)	Overall impact
mCPR (%)	PBF	−0.7	6.6	14.3***	−3.1	7.5	5.7**
	DFF	−1.8	2.4	6.8	4.3	−0.8	3.3
	PBF vs DFF	2.3	2.7	4.7	−3.9	6.9	2.1
Penta3 immunization (%)	PBF	1.2	5.6	10.4	11.6	24.3*	11.1*
	DFF	14.7**	10.4	23.3***	15.5**	29.9**	20.6***
	PBF vs DFF	−12.1	0.1	−7.1	−1.2	−7.4	−6.0*
Institutional delivery (%)	PBF	−3.7	1.6	8.4*	4.4	22.4**	6.7*
	DFF	−5.5	−19.3***	−0.4	−8.9	15.8**	−3.1
	PBF vs DFF	1.7	22.1***	9.2*	14.0***	2.5	10.1***
At least 4 ANC visits (%)	PBF	−1.1*	−29.4***	1.3	0.8	10.6	−3.7
	DFF	−6.8	−24.5***	−1.6	−3.9	14.6**	−3.5
	PBF vs DFF	−7.5	−9.1	−0.01	4.7	−5.6	−2.5
ITN use by under-fives (%)	PBF	−0.7	2.0	−6.5	−7.6	1.2	−0.9
	DFF	1.3	−0.5	4.4	4.2	0.5	3.2
	PBF vs DFF	−2.3	3.7	−11.7**	−14.6**	−0.1	−5.6*

Impact on service utilization indicators, disaggregated by the wealth of the patient. ^*^p<0.1, ^**^p<0.05, and ^***^p<0

The QOC indicators described in the protocol increased significantly relative to the control arm. Of the 26 QOC indicators in the protocol, 21 (81%) adjusted DiDs favored PBF and DFF and 20 (77%) were statistically significant (*p*<0.05). As seen in Table 5, significant improvements were seen in structural QOC such as availability of drugs, equipment, proper handwashing stations, and healthcare waste management. PBF and DFF facilities also carried out much more outreach. On process QOC, the results were more mixed. The proportion of health workers following national protocols for under-five examinations declined (but not as much as in the control arm), and ANC protocol completion improved only a little. Few differences exist between PBF and DFF on QOC, except for an improvement in the availability of basic delivery equipment and contraceptives in PBF facilities. Detailed results as well as those for the other QOC indicators are provided in Tables 5–12 of Additional file 3.

**Table 5:** Impact on QOC indicators

Indicator	Baseline Coverage			Change from baseline % points			Quasi-experimental adjusted DiD		Experimental DiD
	Control	PBF	DFF	Control	PBF	DFF	PBF vs. control	DFF vs. control	PBF vs. DFF
% of facilities with one or more female clinical staff present on day of survey	86.3	85.9	82.3	−8.4	7.8	8.2	20.2***	19.0***	0.9
% of health facilities (HFs) with water for hand washing, soap, clean towel in patient area	73.0	73.9	62.2	−4.0	21.8	29.6	38.3***	48.4***	−8.0
% of HFs with basic delivery equipment	15.4	17.5	16.7	−2.0	65.0	51.5	68.8***	51.8***	13.4**
Number of essential drugs available on day of survey (out of 18)	7.5	6.4	6.6	0.7	8.9	7.5	8.5***	7.1***	1.3*
Average number of contraceptive methods in stock on the day of survey	1.8	1.4	1.4	0.1	1.6	1.2	1.7***	1.2***	0.4**
% of HFs that have a working waste disposal system (bin, pit, or incinerator) in use & safety box for sharps	66.0	64.3	63.3	4.0	32.1	39.4	33.6***	35.4***	−7.4
Average health worker clinical knowledge score (maximum 100)	46.1	47.6	44.3	−8.6	7.3	−3.8	2.4	5.3	−0.04
Under-five examination quality score (based on IMCI protocols; maximum 100)	63.9	46.4	43.6	−21.0	2.1	6.5	5.3	10.8**	−0.04
ANC examination quality score (based on national ANC protocols; maximum 100)	63.8	59.6	47.4	−21.0	−11.0	−3.9	11.3**	17.4***	−0.08
% of HFs that conduct outreach for key MCH services	35.3	45.3	52.3	−5.8	25.8	19.1	25.1***	19.0***	7.2

Impact of the program on quality of care provided at treated health facilities. ^*^p<0.1, ^**^p<0.05, and ^***^p<0.01

## Discussion

This study demonstrates that both PBF and DFF had important effects on the coverage and structural quality of MCH services. Specifically, we find that PBF is superior to DFF as for institutional deliveries and some measures of quality improvement, but with no incremental impacts for other examined indicators. We find some limited correlation of the program impact with coverage rates at the baseline, which suggests caution for interpretation. Nonetheless, both PBF and DFF present significant improvements over business-as-usual. In the Nigerian context, where there has been limited progress in population coverage of these services over the last 25 years, and indeed some worsening of outcomes seen in the 2018 DHS, the improvements seen in these treatment arms are significant and encouraging. Under real-world conditions and at a large scale, PBF and DFF appear to be effective and practical interventions. Our findings thus provide evidence on two interventions both of which leverage the benefits of directing funding to PHC facilities paired with autonomy, community engagement, and strengthened supervision as a policy option in Nigeria and countries that face similar PHC issues. Indeed, the DFF secures many mechanism cores to PBF, including direct transfer on health facilities’ bank accounts, autonomy of the health facility in the management of these funds, and enhanced supervision, but skips on the intensive and expensive monitoring and enforcement systems. While we do not find that the specific type of PBF studied had an additional effect except for institutional delivery, we note that the institutional delivery impact is large: indeed, it is even larger than that reported in a seminal paper on the impact of PBF on institutional deliveries in Rwanda [10]. It is also noteworthy that the effect sizes for the process quality outcomes (under-5 and ANC examination scores, and percent of health facilities conducting outreach) range from 5.3 to 25.1 percentage points. These effects are comparable to the interquartile range of effects of supervision as a standalone intervention from a systematic review of 16 study comparisons of supervision: 6.2 to 25.2 percentage points [49]. In other words, the observed effects on the quality of care may in large part arise from the supervision component of the PBF and DFF interventions.

This study is not without caveats. While PBF and DFF were randomly assigned at the district level, treated states were purposively chosen. Thus, we adjust the DiD comparison of treatment and control facilities. Further, while PBF and DFF are comprehensive intervention packages that include devolved autonomy and enhanced monitoring, supervision, data verification, and community engagement, this study was not able to assess the relative effectiveness of each of these components but rather the summary effect of all. Further investigation may consider the impacts of each of these components.

The study also faced some methodological challenges, in particular external validity may be reduced by the fact that treatment states were purposively chosen, and households were selected from the catchment areas of facilities and not from the district. Further, treatment assignment at a relatively large administrative unit (district) may mitigate potential contamination effects, but likely does not entirely rule it out. It may thus be the case that DFF facilities performed especially well because they believed that high performance would lead to their receiving the PBF bonuses. Finally, while spillovers from the PBF checklist onto unincentivized services may be a concern, the Nigeria State Health Investment Project PBF intervention was designed with potential negative spillovers on unincentivized services in mind. It thus purchased the 20 most common services provided by PHC facilities, thereby leaving few by which to judge such unintended consequences. On the other hand, the study benefits from more than 3 years of program exposure, while most comparable studies look at impacts of PBF after 1.5–2 years [10, 34, 35, 50–52]. The intervention was also extensive in size and involved 9.5 million people (3.6 million in Adamawa, 1.9 million in Nasarawa, and 4 million in Ondo) living in 52 districts and covered more than 1389 PHC facilities. The scale of implementation, duration of exposure to the intervention, and modest levels of incremental funding likely mitigate the pilot test bias and contribute to external validity.

Our results may shed some light on potential mechanisms. While randomization to PBF or DFF arms resulted in a balanced set of districts, in a few instances, baseline coverage levels for DFF are (generally insignificantly) lower and the DFF treatment impacts are correspondingly the highest. This pattern may suggest that the DFF impacts are large because of the baseline levels happened to be relatively low. We note, however, that this is not always the case; for example, the impact estimates for mCPR are greater for PBF than DFF although the baseline coverage is lowest for DFF. While this study intentionally does not address this and other contextual factors, evidence suggests that local context may drive the success of health financing—and indeed many other—interventions. Such contextual factors might include the PFM structure, health worker background, local norms around MCH service utilization, and of course pre-existing health system capacity. Indeed, the context has been shown to be an important driver of PBF program success, including in Nigeria [53, 54].

A costing analysis examined the financial costs rather than economic costs and included costs for program implementation and verification, and donor supervision [55]. It finds that the all-in cost, from a government absorptive capacity or health system perspective, to implement PBF was US$3.49 per capita per year, while DFF cost US$1.74 per capita per year over the 5-year lifespan of the program. Note that while these are 5-year costs, we evaluate the first 3 years of implementation of the program, which happened after all the necessary systems had been put in place. The financial audit revealed that (i) SPHCDAs arranged for the transfer of the correct amount of funds to each facility, and the average payment was accomplished in 51 days (compared to the 45-day standard established at the beginning of the study); (ii) there was no evidence of “phantom” health facilities receiving funds; (iii) PBF and DFF funds accounted for about 95% of disposable funds—that is, operational expenses, after other recurring expenditures—available in the PBF and DFF facilities and were being used appropriately to meet operational expenses; (iv) financial management in PBF and DFF facilities needed to be improved, as some expenses were not recorded properly, vendors were sometimes paid in cash, and in some facilities, the system of signatories was not being followed [55]. The risks related to providing funds directly to facilities are mitigated by community engagement and strengthened supervision. In addition, because facility-based staff are client-facing and can be scrutinized by the community, PBF and DFF may face a lower risk of corruption than funds spent at higher levels.

While the PBF and DFF interventions offer several encouraging results, three important issues remain: (i) the endline coverage of MCH services remains mediocre by comparison to Nigeria’s neighbors. For example, the endline coverage of Penta3 immunization, at 56.4%, is still lower than the greater-than-80% coverage achieved in Cameroon, Ghana, and Senegal [56–58]; (ii) process measures of QOC did not improve systematically in the PBF arm over the DFF arm; and (iii) usage by the poorest did not improve as much as expected. This last finding is consistent with the finding in other work that wealthier patients are quality sensitive [59, 60]. Consistent with evidence, we find both interventions improve structural quality but these improvements do not translate into changes in clinical or process quality [61]. However, we also find that both PBF and DFF improve the quality relative to business-as-usual, which is consistent with the finding from some studies that PBF can improve clinical quality [34]. These challenges suggest that strengthening health facility management to take full advantage of the resources and autonomy provided under PBF and DFF requires further exploration. The impact of a PBF or DFF type intervention may be further strengthened by adding on other non-monetary incentives for improving health worker performance (e.g., group problem-solving, information, and communication technology) may lead to improvements in service delivery [49]. Finally, the literature highlights that the impacts of supply-side interventions, including health facility financing, may be bolstered by other approaches such as demand-side efforts to increase usage by the poorest.

## Conclusions

A central criticism of PBF interventions pertains to their complexity of design and implementation and their donor-driven backing [22, 23, 62]. Indeed, a systematic review of interventions designed to improve provider effort finds that simpler interventions work better than multifaceted ones [50]. In this vein, the findings from this study perhaps indicate that PBF and DFF should be viewed as complements and not alternatives: countries may be able to use a very simple health facility financing approach as presented by DFF in our context. In addition, they may either modify the nature of the financial incentive under the PBF approach or target it only to specific indicators such as institutional delivery may yield greater impact with higher efficiency. Specific indicators for PBF may be chosen based on whether they are in the health worker’s locus of control. For instance, some evidence suggests that health workers cannot fully respond to incentives for antenatal care visits because of demand side barriers. On the other hand, services like delivery may be more responsive to health worker effort than antenatal care because health workers can use quality of care during antenatal care to bring a woman back for delivery [10, 12].

Among the appealing aspects of DFF is its relative simplicity of implementation, low administrative cost, and lower risk of gaming when compared with PBF, with no measurement or verification visits required. This aspect may be particularly salient in responding to the health system challenges imposed by COVID-19. While the use of DFF could be widened, there is still much room for innovation and further improvements in the approach. A remaining conundrum relates to the specific design of the payment formula. Finally, and perhaps most critically, this study identifies two health financing options for countries to choose from. Both PBF and DFF offer viable improvements over business-as-usual in moving forward with the desired transformation of health systems. Indeed, the study potentially suggests a way of combining two health financing mechanisms—decentralized financing, autonomy, and monitoring with strategic purchasing for key services.

## Supplementary Information

**Additional file 1: **Calculation of PBF Payments. **Table 1.** Example of PBF in a Health Facility.

**Additional file 2: **Parallel Trends and Balance. **Table 1.** Pre-Intervention Trends in Key Household Characteristics. **Table 2.** Differences in Key Household Characteristics at the Baseline. **Table 3.** Differences in Key Household Characteristics at the Baseline (PBF versus DFF).

**Additional file 3: **Supplementary Results. **Table 1.** Impact on Outcomes of Quality of Care, Maternal and Child Health. **Table 2a.** Impact on Access to Healthcare. **Table 2b.** Impact on Utilization of Healthcare. **Table 3.** Impact on Skilled Birth Attendance and Institutional Delivery, by Wealth. **Table 4.** Impact on ANC and Immunization, by Wealth. **Table 5.** Impact on Structural Quality of Care, Equipment. **Table 6.** Impact on Structural Quality of Care, Drugs. **Table 7.** Impact on Structural Quality of Care, Staffing. **Table 8.** Impact on Structural Quality of Care, Recordkeeping. **Table 9.** Impact on Structural Quality of Care, Sanitation. **Table 10.** Impact on Structural Quality of Care, Tuberculosis. **Table 11.** Impact on Procedural Quality of Care. **Table 12.** Impact on Outcomes of Quality of Care, Interactions at Health Facilities.

## Data Availability

The data and materials are available from the World Bank’s microdata catalog. Kandpal, E. Nigeria State Health Investment Project: Impact Evaluation Endline Survey. World Bank https://microdata.worldbank.org/index.php/catalog/4042 (2017).

## Abbreviations

ANC: Antenatal care
CPR: Contraceptive prevalence rate
DFF: Direct facility financing
DHS: Demographic and Health Survey
ITN: Insecticide-treated mosquito net
LGA: Local governance area
MCH: Maternal and child healthcare
mCPR: Modern contraceptive prevalence rate
NHREC: National Health Research Ethics Committee of Nigeria
PBF: Performance-based financing
PFM: Public finance management
PMC: Primary health care
pps: Percentage points
QOC: Quality of care
QSC: Quantified Supervisory Checklist
SPHCDA: State primary health care development agencies
UHC: Universal health care
WDC: Ward development committee
