# Supplementary material for: Decentralized facility financing versus performance-based payments in primary health care: a large-scale randomized controlled trial in Nigeria
Source: BMC Med. 2021 Sep 21;19:224. doi: 10.1186/s12916-021-02092-4 (PMC8452448; doi:10.1186/s12916-021-02092-4)
Supplement: Supplementary file 1 — Additional file 1: Calculation of PBF Payments. Table 1. Example of PBF in a Health Facility. [file 12916_2021_2092_MOESM1_ESM.docx]

**Additional file 1: Calculation of PBF Payments**

Table 1 describes an example of the how payments to a given health facility were calculated under PBF. In this example, if a health facility fully immunizes 50 children in a quarter, they could earn US$100 (100 x US$2 per child fully vaccinated). Under NSHIP, 20 specific services were incentivized in primary health facilities. In this example the facility would have earned $1,600. The total would have to be adjusted by a quality score based on a quantitative checklist administered at the facility every quarter. This facility would have earned 50 percent times 25 percent of its quantity payment, i.e. $200. Money would be transferred electronically to the facility’s bank account. Facilities could use these funds for: (i) health facility operational costs (about 50%), including maintenance and repair, drugs and consumables, outreach and other quality enhancement measures; and (ii) performance bonus for health workers (up to 50%). To incentivize improvements in quality of care at the secondary level, including referral from primary health facilities, the project tested a similar PBF approach in secondary hospitals. However, given the small number of secondary facilities and the focus on primary health care, this IE focuses on results at the primary health facility level.

Table 1: Example of PBF in a Health Facility

| Service | Number Provided  Last Quarter | Unit Price | Total Earned |
| --- | --- | --- | --- |
| Child fully vaccinated | 50 | US$2 | US$100 |
| Skilled birth attendance | 60 | US$10 | US$600 |
| Curative care patient visit | 1,800 | US$0.5 | US$900 |
| Sub-Total |  |  | US$1,600 |
| Quality bonus | Score (50%) x 25% of volume | | US$200 |
| Total | | | US$1,800 |
| Use of Funds | | |  |
| Drugs and consumables | | | US$400 |
| Outreach expenditures | | | US$150 |
| Repairs & maintenance of health facility | | | US$150 |
| Bonuses to staff in the facility | | | US$900 |
| Savings | | | US$200 |
