# Supplementary material for: Decentralized facility financing versus performance-based payments in primary health care: a large-scale randomized controlled trial in Nigeria
Source: BMC Med. 2021 Sep 21;19:224. doi: 10.1186/s12916-021-02092-4 (PMC8452448; doi:10.1186/s12916-021-02092-4)
Supplement: Supplementary file 2 — Additional file 2: Parallel Trends and Balance. Table 1. Pre-Intervention Trends in Key Household Characteristics. Table 2. Differences in Key Household Characteristics at the Baseline. Table 3. Differences in Key Household Characteristics at the Baseline (PBF versus DFF). [file 12916_2021_2092_MOESM2_ESM.docx]

**Additional file 2: Parallel Trends and Balance**

The double-difference method uses the trend in the comparison LGAs as an estimate of the counterfactual for the trend in the treatment LGAs. The validity of this approach crucially depends on the assumption that the average change in the comparison LGAs reflects the counterfactual change in the treatment LGAs if there were no treatment. This is called the *parallel trends assumption*. In the context of this study, the assumption of parallel trends implies that the trends in relevant outcomes across project and treatment states were the same before NSHIP started and can be tested using Equation 1 on pre-NSHIP data. If the assumption of parallel trends holds, the estimates of $\beta_{3}$ will be insignificant. Table 5 describes this test for socio-economic indicators available from the Nigerian DHS of 2008 and 2013. Although most of the indicators change at the same rate in the absence of the project between 2008 and 2013, some key outcomes like skilled birth attendance rate do not exhibit parallel trends across treatment and control states. To summarize, out of the twenty-two key household characteristics explored, parallel trends do not hold for eight. However, it is not always the case that control states are worse off than treatment states; in only one out of these twenty-two comparisons, control states higher rates of improvement. If the control states were always worse off than the treatment states, we would worry about their validity as counterfactuals. A comparison group that is consistently worsening would suggest that we were overestimating the impact of the program by comparing it to an artificially low counterfactual.

Table 1: Pre-Intervention Trends in Key Household Characteristics

|  | Year 2013 | Project States | Year 2013*Project States ($\boldsymbol{\beta}_{\boldsymbol{3}}\boldsymbol{)}$ |
| --- | --- | --- | --- |
| If any child (younger than 24 months) in the HH ever received any vaccination | 0.050 | -0.081** | -0.069 |
|  | (0.034) | (0.035) | (0.047) |
| If the mother (of at least one child younger than 24 months) received skilled assistance during child birth | -0.002 | -0.197*** | 0.129** |
|  | (0.041) | (0.040) | (0.056) |
| If the mother (of at least one child younger than 24 months) received 4 or more ANC visits | 0.024 | 0.020 | 0.062 |
|  | (0.036) | (0.036) | (0.050) |
| If the child (younger than 24 months) slept under an ITN the night before the survey | 0.089*** | 0.001 | -0.018 |
|  | (0.011) | (0.006) | (0.014) |
| If any child's birth in the HH has been registered | -0.010 | -0.035** | 0.063*** |
|  | (0.016) | (0.016) | (0.023) |
| If child in the HH is stunted | -0.022 | -0.033** | 0.035* |
|  | (0.015) | (0.014) | (0.021) |
| If child in the HH is wasted | 0.007 | 0.011 | 0.004 |
|  | (0.006) | (0.010) | (0.012) |
| If child in the HH is underweight | 0.028** | 0.007 | -0.002 |
|  | (0.013) | (0.012) | (0.018) |
| Household has a literate woman | -0.011 | -0.030 | 0.062 |
|  | (0.030) | (0.035) | (0.049) |
| Household has a woman who can read a newspaper | -0.034*** | -0.021* | 0.067*** |
|  | (0.011) | (0.011) | (0.017) |
| Household has an employed woman | -0.043* | -0.125*** | 0.042 |
|  | (0.025) | (0.026) | (0.038) |
| Women in the household use modern contraceptive methods | 0.026* | -0.014 | -0.008 |
|  | (0.016) | (0.016) | (0.024) |
| Household has a man who can read | 0.025 | 0.077*** | -0.066* |
|  | (0.023) | (0.026) | (0.038) |
| Proportion of women who received skilled post-natal care (PNC) | 0.047 | 0.043 | 0.002 |
|  | (0.033) | (0.034) | (0.044) |
| Proportion of women who received tetanus before birth | -0.038 | -0.067** | 0.121*** |
|  | (0.029) | (0.032) | (0.041) |
| Proportion of child births at an institution | -0.027 | -0.164*** | 0.110** |
|  | (0.035) | (0.031) | (0.043) |
| Fever prevalence rate | -0.031 | -0.057*** | 0.053** |
|  | (0.021) | (0.016) | (0.024) |
| Diarrhea prevalence rate | 0.031* | -0.036** | 0.012 |
|  | (0.017) | (0.014) | (0.022) |
| Proportion of households that have at least one ITN | 0.519*** | 0.020 | 0.028 |
|  | (0.029) | (0.013) | (0.036) |
| Proportion of households with improved water source | -0.030 | -0.021 | 0.054 |
|  | (0.046) | (0.046) | (0.065) |
| Proportion of households with improved sanitation | 0.009 | 0.030 | -0.057 |
|  | (0.033) | (0.034) | (0.050) |
| Proportion of households that have electricity | -0.060* | -0.010 | 0.028 |
|  | (0.035) | (0.038) | (0.056) |
| Clustered standard errors (at LGA level) in parentheses |  |  |  |
| * p<0.1, ** p<0.05, *** p<0.01 |  |  |  |
| Source: Authors’ calculations using DHS 2008 and 2013 data |  |  |  |

We also compare these indicators, and some additional variables that capture quality and quantity of healthcare using the baseline data we collected for the purposes of this evaluation. However, with the matched states design, we cannot establish balance at the baseline on several important variables (Tables 6). To allay any resulting concerns about the validity of the control comparison, we use the variables initially used to compute the Euclidian distance between the project and the control states to generate a propensity that a PSU falls in either a treatment or a control state. Following Hirano, Imbens & Ridder (2003), we then use this predicted probability to reweight the estimates obtained from equation 1.^^[[1]](#footnote-1)^^ This reweighting process thus assigns a greater weight to the observations that are in the project states but should have been in control states. Units that were expectedly placed in the project states are assigned a lower weight because a lot of information already available on such units.

Table 2: Differences in Key Household Characteristics at the Baseline

| Variable | Comparison States | NSHIP States | Difference |
| --- | --- | --- | --- |
| Female employment rate | 0.656 | 0.57 | 0.086*** |
|  | (0.017) | (0.011) |  |
| Proportion of children who have received all basic vaccination | 0.234 | 0.299 | -0.066*** |
|  | (0.014) | (0.012) |  |
| Fever prevalence rate | 0.043 | 0.027 | 0.016*** |
|  | (0.006) | (0.002) |  |
| Diarrhoea prevalence rate | 0.015 | 0.011 | 0.004 |
|  | (0.002) | (0.001) |  |
| % of children 6-23 months old who received BCG | 0.64 | 0.657 | -0.017 |
|  | (0.017) | (0.013) |  |
| % of children (12-23mths) who receive Penta3 | 0.332 | 0.391 | -0.059** |
|  | (0.017) | (0.014) |  |
| Under-5 curative care expenditure | 252.032 | 218.279 | 33.753 |
|  | (24.355) | (17.083) |  |
| Average years of completed education | 6.31 | 6.945 | -0.752** |
|  | (0.231) | (0.172) |  |
| Proportion of households with improved water source | 0.394 | 0.353 | 0.055** |
|  | (0.020) | (0.014) |  |
| Proportion of households with improved sanitation | 0.174 | 0.192 | -0.027 |
|  | (0.016) | (0.012) |  |
| Proportion of households that have electricity | 0.252 | 0.346 | -0.075** |
|  | (0.023) | (0.018) |  |
| Female literacy | 0.446 | 0.49 | -0.060** |
|  | (0.020) | (0.013) |  |
| Household has a woman who can read a newspaper | 0.156 | 0.147 | 0.018* |
|  | (0.011) | (0.007) |  |
| Average number of children in a household | 3.579 | 3.658 | -0.064 |
|  | (0.066) | (0.046) |  |
| Proportion of women who received skilled PNC | 0.214 | 0.216 | 0.016 |
|  | (0.013) | (0.010) |  |
| Proportion of births attended by skilled personnel | 0.641 | 0.577 | 0.066*** |
|  | (0.020) | (0.014) |  |
| Proportion of institutional Delivery | 0.572 | 0.507 | 0.048*** |
|  | (0.020) | (0.014) |  |
| Proportion of women who received tetanus before child birth | 0.461 | 0.571 | -0.106*** |
|  | (0.017) | (0.012) |  |
| Proportion of households that have at least one ITN | 0.636 | 0.707 | -0.066*** |
|  | (0.019) | (0.011) |  |
| Proportion of children who received IPT2 vaccines | 0.097 | 0.173 | -0.064*** |
|  | (0.008) | (0.009) |  |
| Women in the household use modern contraceptive methods | 0.204 | 0.169 | 0.010* |
|  | (0.013) | (0.009) |  |
| Clustered standard errors (at EA level) in parentheses |  |  |  |
| * p<0.1, ** p<0.05, *** p<0.01 |  |  |  |

Since LGA assignment to PBF or DFF arms was randomized, and was largely successful in ensuring that the averages of key household characteristics do not differ across these project arms (Table 7), we do not reweight these estimates.

Table 3: Differences in Key Household Characteristics at the Baseline (PBF versus DFF)

|  | DFF LGAs | PBF LGAs | Difference |
| --- | --- | --- | --- |
| Median completed years of education | 7.12 | 6.93 | -0.190 |
|  | (0.358) | (0.321) | (0.479) |
| Proportion of households with improved water source | 0.34 | 0.35 | 0.012 |
|  | (0.035) | (0.037) | (0.051) |
| Proportion of households with improved sanitation | 0.20 | 0.18 | -0.021 |
|  | (0.025) | (0.020) | (0.032) |
| Proportion of households that have electricity | 0.31 | 0.35 | 0.041 |
|  | (0.039) | (0.047) | (0.060) |
| Female literacy | 0.49 | 0.50 | 0.006 |
|  | (0.023) | (0.021) | (0.031) |
| Household has a woman who can read a newspaper | 0.14 | 0.14 | 0.001 |
|  | (0.010) | (0.016) | (0.019) |
| Average number of children in the household | 3.54 | 3.76 | 0.222** |
|  | (0.075) | (0.074) | (0.105) |
| Proportion of women who received skilled PNC | 0.24 | 0.19 | -0.046 |
|  | (0.024) | (0.023) | (0.033) |
| Proportion of women who received tetanus before child birth | 0.57 | 0.56 | -0.017 |
|  | (0.029) | (0.031) | (0.043) |
| Proportion of child births at an institution | 0.52 | 0.47 | -0.050 |
|  | (0.030) | (0.034) | (0.045) |
| Proportion of households that have at least one ITN | 0.67 | 0.76 | 0.094*** |
|  | (0.022) | (0.021) | (0.031) |
| Proportion of children who received IPT2 vaccines | 0.17 | 0.19 | 0.013 |
|  | (0.024) | (0.029) | (0.038) |
| Women in the household use modern contraceptive methods | 0.16 | 0.18 | 0.018 |
|  | (0.013) | (0.015) | (0.020) |
| Female employment rate | 0.59 | 0.55 | -0.035 |
|  | (0.021) | (0.028) | (0.035) |
| Proportion of children who have received all basic vaccination | 0.24 | 0.37 | 0.124** |
|  | (0.035) | (0.034) | (0.049) |
| Fever prevalence rate | 0.02 | 0.03 | 0.009 |
|  | (0.006) | (0.008) | (0.010) |
| Diarrhea prevalence rate | 0.01 | 0.01 | 0.006 |
|  | (0.002) | (0.003) | (0.004) |
| Clustered standard errors (at EA level) in parentheses |  |  |  |
| * p<0.1, ** p<0.05, *** p<0.01 |  |  |  |

- 1. **Sampling**

One health center in each ward of each sampled LGA was selected to be a part of the sample. In addition, three health workers were interviewed at each facility. Households were chosen in the following way. The National Population Commission of Nigeria listed all enumeration areas in the country for the 2006 census of Nigeria. In 2008, the Federal Ministry of Health used these enumeration areas to create facility catchment areas. These were our PSUs. The power calculation results determined that the number of PSUs per site is 17 for Nasarawa; 12 for Ondo; 10 for Adamawa; 17 for Benue; 12 for Ogun; and 10 for Taraba.

After determining the number of PSUs per state, the next step is to select sample PSUs. Each EA has some conspicuous natural or man-made features as boundaries. During EA delineation, states and LGAs were canvassed in geographical order, from west to east and back to the west in a serpentine fashion. The catchment areas within the LGAs are thus contiguous so that no one catchment area boundary overlaps the other and no area is omitted. The catchment areas were coded serially one after the other using map orientation in a serpentine order. From this list, PSUs were randomly selected on a probability proportional to size basis.

The last step entailed the selection of households. A household listing exercise was carried out in every PSU. The listing form was designed to obtain information about the address of buildings, number of households in the listed buildings, name of the head of each household listed, and presence of woman in the household with at least one pregnancy or birth in the two years preceding the survey. Using this list as the sampling frame, 15 households with a woman who had experienced at least one pregnancy during the last two years were selected from every PSU. If a PSU had fewer than 15 households, the listing exercise extended into the contiguous PSU.

Note that inference is somewhat limited by the representativeness of our results: given that project states were purposively chosen and that households were selected from the catchment areas of our study facilities, our results are representative of the project at the state level, and not of the entire country or even state.

1. For units in project states, weight is equal to the inverse of the predicted probability and for units in comparison states, this weight is equal to the inverse of (1-predicted probability). [↑](#footnote-ref-1)
