# Supplementary material for: Decentralized facility financing versus performance-based payments in primary health care: a large-scale randomized controlled trial in Nigeria
Source: BMC Med. 2021 Sep 21;19:224. doi: 10.1186/s12916-021-02092-4 (PMC8452448; doi:10.1186/s12916-021-02092-4)
Supplement: Supplementary file 3 — Additional file 3: Supplementary Results. Table 1. Impact on Outcomes of Quality of Care, Maternal and Child Health. Table 2a. Impact on Access to Healthcare. Table 2b. Impact on Utilization of Healthcare. Table 3. Impact on Skilled Birth Attendance and Institutional Delivery, by Wealth. Table 4. Impact on ANC and Immunization, by Wealth. Table 5. Impact on Structural Quality of Care, Equipment. Table 6. Impact on Structural Quality of Care, Drugs. Table 7. Impact on Structural Quality of Care, Staffing. Table 8. Impact on Structural Quality of Care, Recordkeeping. Table 9. Impact on Structural Quality of Care, Sanitation. Table 10. Impact on Structural Quality of Care, Tuberculosis. Table 11. Impact on Procedural Quality of Care. Table 12. Impact on Outcomes of Quality of Care, Interactions at Health Facilities. [file 12916_2021_2092_MOESM3_ESM.docx]

**Additional file 3: Supplementary Results**

Table 1: Impact on Outcomes of Quality of Care, Maternal and Child Health

|  | PBF  vs  Control | DFF  vs  Control | PBF  vs  DFF | Baseline Mean | N |
| --- | --- | --- | --- | --- | --- |
| Proportion of women who use modern contraceptive methods | 0.057** | 0.033 | 0.021 | 0.17 | 13,448 |
|  | (2.15) | (1.24) | (1.00) |  |  |
| Proportion of children (12-23 months) who receive Penta3 | 0.111*** | 0.206*** | -0.060* | 0.39 | 7,437 |
|  | (3.01) | (5.73) | (1.69) |  |  |
| Proportion of institutional Delivery | 0.067* | -0.03 | 0.101*** | 0.53 | 14,566 |
|  | (1.86) | (0.83) | (2.95) |  |  |
| Proportion of pregnant women receiving 4 or more ANC visits | -0.038 | -0.035 | -0.025 | 0.50 | 14,913 |
|  | (1.10) | (1.06) | (0.69) |  |  |
| Proportion of children under 5 who slept under an ITN last night | -0.009 | 0.032 | -0.056* | 0.45 | 22,441 |
|  | (0.30) | (1.07) | (1.94) |  |  |
| Curative care expenditure on children under 5 (in Naira) | 105.788 | 150.824 | -116.336 | 229 | 14,418 |
|  | (0.78) | (1.48) | (0.96) |  |  |
| Standard errors clustered at LGA level | | | |  |  |
| t statistics in parentheses |  |  |  |  |  |
| * p<0.1, ** p<0.05, *** p<0.01 |  |  |  |  |  |

Table 2a: Impact on Access to Healthcare

|  | PBF  vs  Control | DFF  vs  Control | PBF  vs  DFF | Baseline Mean | | N | |
| --- | --- | --- | --- | --- | --- | --- | --- |
| % of health facilities that offered routine immunizations in the week of the survey | 0.132* | 0.069 | 0.087 | 0.62 | | 1490 | |
|  | (1.71) | (0.71) | (1.10) |  | |  | |
| % of health facilities that offer delivery services in facility or skilled birth attendance in the community | 0.079 | 0.101 | -0.03 | 0.94 | | 1515 | |
|  | (1.06) | (1.37) | (1.10) |  | |  | |
| Standard errors clustered at LGA level | |  |  | |  | |  |
| t statistics in parentheses |  |  |  | |  | |  |
| * p<0.1, ** p<0.05, *** p<0.01 |  |  |  | |  | |  |

Table 2b: Impact on Utilization of Healthcare

|  | PBF  Vs  Control | DFF  Vs  Control | PBF  Vs  DFF | Baseline Mean | | N | |
| --- | --- | --- | --- | --- | --- | --- | --- |
| Number of facility deliveries or deliveries in communities attended by skilled personnel in the 30 days preceding the survey | 14.813*** | 9.415*** | 4.972 | 22.8 | | 1516 | |
|  | (6.02) | (3.24) | (1.48) |  | |  | |
| Number of ANC visits in the 30 days preceding the survey | 48.235*** | 45.614*** | 4.331 | 34.2 | | 1526 | |
|  | (3.62) | (3.71) | (0.42) |  | |  | |
| Number of immunizations offered in the 30 days preceding the survey | 469.31 | 509.792* | -41.357 | 272.6 | | 1490 | |
|  | (1.64) | (1.76) | (0.61) |  | |  | |
| Number of curative care visits from children aged under five in the 30 days preceding the survey | 45.300*** | 39.311*** | 7.112 | 28.6 | | 1526 | |
|  | (4.16) | (2.85) | (0.46) |  | |  | |
| Standard errors clustered at LGA level | |  |  | |  | |  |
| t statistics in parentheses |  |  |  | |  | |  |
| * p<0.1, ** p<0.05, *** p<0.01 |  |  |  | |  | |  |

Table 3: Impact on Skilled Birth Attendance and Institutional Delivery, by Wealth

| PBF |  |  |  |  |  |
| --- | --- | --- | --- | --- | --- |
|  | **Q1** | **Q2** | **Q3** | **Q4** | **Q5** |
| Proportion of births attended by skilled personnel | -0.025 | 0.045 | 0.118** | 0.110** | 0.061 |
|  | (0.36) | (0.73) | (2.12) | (2.07) | (1.15) |
| Proportion of institutional delivery | -0.038 | -0.036 | 0.082 | 0.128** | 0.069 |
|  | (0.61) | (0.60) | (1.45) | (2.22) | (1.24) |
| DFF |  |  |  |  |  |
|  | **Q1** | **Q2** | **Q3** | **Q4** | **Q5** |
| Proportion of births attended by skilled personnel | -0.012 | -0.092 | 0.007 | -0.065 | 0.002 |
|  | (0.19) | (1.49) | (0.13) | (1.20) | (0.03) |
| Proportion of institutional delivery | -0.062 | -0.115* | -0.049 | -0.083 | 0.038 |
|  | (0.95) | (1.80) | (0.84) | (1.42) | (0.62) |
| PBF vs DFF | |  |  |  |  |
|  | **Q1** | **Q2** | **Q3** | **Q4** | **Q5** |
| Proportion of births attended by skilled personnel | 0.007 | 0.111* | 0.142*** | 0.185*** | 0.082* |
|  | (0.12) | (1.83) | (2.78) | (3.98) | (1.87) |
| Proportion of institutional delivery | 0.023 | 0.079 | 0.156*** | 0.209*** | 0.027 |
|  | (0.38) | (1.26) | (3.00) | (4.25) | (0.59) |
| Standard errors clustered at LGA level |  |  |  |  |  |
| t statistics in parentheses |  |  |  |  |  |
| * p<0.1, ** p<0.05, *** p<0.01 |  |  |  |  |  |

Table 4: Impact on ANC and Immunization, by Wealth

| PBF |  |  |  |  |  |
| --- | --- | --- | --- | --- | --- |
|  | **Q1** | **Q2** | **Q3** | **Q4** | **Q5** |
| Proportion of pregnant women receiving 4 or more ANC visits | -0.152** | -0.18*** | -0.082 | -0.029 | 0.073 |
|  | (2.41) | (2.92) | (1.42) | (0.53) | (1.32) |
| Proportion of children (12-23 months) fully immunized | 0.035 | 0.064 | 0.003 | 0.138* | 0.181** |
|  | (0.46) | (1.01) | (0.05) | (1.81) | (2.21) |
| Proportion of children (12-23 months) who receive Penta3 | 0.083 | 0.075 | -0.021 | 0.099 | 0.207*** |
|  | (0.98) | (1.11) | (0.29) | (1.28) | (2.61) |
| DFF |  |  |  |  |  |
|  | **Q1** | **Q2** | **Q3** | **Q4** | **Q5** |
| Proportion of pregnant women receiving 4 or more ANC visits | -0.058 | -0.17*** | -0.097* | -0.079 | 0.086 |
|  | (0.98) | (2.97) | (1.67) | (1.44) | (1.59) |
| Proportion of children (12-23 months) fully immunized | 0.105 | 0.074 | 0.08 | 0.204*** | 0.249*** |
|  | (1.49) | (1.16) | (1.10) | (2.71) | (2.96) |
| Proportion of children (12-23 months) who receive Penta3 | 0.199*** | 0.123* | 0.126* | 0.148* | 0.275*** |
|  | (2.74) | (1.74) | (1.69) | (1.90) | (3.29) |
| PBF vs DFF |  |  |  |  |  |
|  | **Q1** | **Q2** | **Q3** | **Q4** | **Q5** |
| Proportion of pregnant women receiving 4 or more ANC visits | -0.097 | -0.049 | 0.004 | 0.016 | -0.015 |
|  | (1.61) | (0.81) | (0.07) | (0.33) | (0.31) |
| Proportion of children (12-23 months) fully immunized | -0.041 | 0.00 | -0.034 | -0.024 | -0.064 |
|  | (0.54) | (0.00) | (0.52) | (0.40) | (1.00) |
| Proportion of children (12-23 months) who receive Penta3 | -0.101 | -0.035 | -0.086 | -0.027 | -0.049 |
|  | (1.21) | (0.50) | (1.23) | (0.42) | (0.76) |
| Standard errors clustered at LGA level |  |  |  |  |  |
| t statistics in parentheses |  |  |  |  |  |
| * p<0.1, ** p<0.05, *** p<0.01 |  |  |  |  |  |

Table 5: Impact on Structural Quality of Care, Equipment

|  | PBF  vs  Control | DFF  vs  Control | PBF  vs  DFF | Baseline Mean | | N | |  |
| --- | --- | --- | --- | --- | --- | --- | --- | --- |
| Proportion of health facilities with basic delivery equipment | 0.688*** | 0.518*** | 0.134** | 0.17 | | 1516 | |  |
|  | (12.06) | (8.22) | (2.06) |  | |  | |  |
| Proportion of health facilities with basic ANC equipment | 0.340*** | 0.342*** | -0.037 | 0.49 | | 1526 | |  |
|  | (3.87) | (4.54) | (0.70) |  | |  | |  |
| Proportion of health facilities with basic routine immunization equipment | 0.074 | -0.077 | 0.158** | 0.09 | | 1490 | |  |
|  | (0.93) | (0.94) | (2.14) |  | |  | |  |
| Proportion of health facilities with basic clinical equipment | 0.129*** | 0.135*** | 0.003 | 0.06 | | 1526 | |  |
|  | (3.24) | (4.48) | (0.08) |  | |  | |  |
| Standard errors clustered at LGA level | | | | |  | |  | |
| t statistics in parentheses |  |  |  | |  | |  | |
| * p<0.1, ** p<0.05, *** p<0.01 |  |  |  | |  | |  | |

Table 6: Impact on Structural Quality of Care, Drugs

|  | PBF  vs  Control | DFF  vs  Control | PBF  vs  DFF | Baseline Mean | N | |  |
| --- | --- | --- | --- | --- | --- | --- | --- |
| Number of essential drugs available on the day of the survey | 8.490*** | 7.114*** | 1.290* | 6.8 | 1526 | |  |
|  | (11.16) | (10.53) | (1.73) |  |  | |  |
| Average number of contraceptive methods in stock on the day of survey | 1.729*** | 1.240*** | 0.426*** | 1.5 | 1526 | |  |
|  | (8.00) | (6.12) | (2.91) |  |  | |  |
| Proportion of health facilities with bednets in stock on the day of the survey | 0.486*** | 0.487*** | 0.006 | 0.30 | 1526 | |  |
|  | (4.78) | (4.37) | (0.06) |  |  | |  |
| Standard errors clustered at LGA level |  |  |  |  | |  | |
| t statistics in parentheses |  |  |  |  | |  | |
| * p<0.1, ** p<0.05, *** p<0.01 |  |  |  |  | |  | |

Table 7: Impact on Structural Quality of Care, Staffing

|  | PBF  vs  Control | DFF  vs  Control | PBF  vs  DFF | | Baseline Mean | | | N | | |  |
| --- | --- | --- | --- | --- | --- | --- | --- | --- | --- | --- | --- |
| Proportion of on-duty technical staff present at health facility on the day of survey | -0.021 | -0.1 | 0.063* | | 0.50 | | | 1411 | | |  |
|  | (0.31) | (1.37) | (1.97) | |  | | |  | | |  |
| At least one female clinical staff present on the day of survey | 0.202** | 0.190** | 0.009 | | 0.84 | | | 1505 | | |  |
|  | (2.16) | (2.01) | (0.24) | |  | | |  | | |  |
| Proportion of health workers who report receiving their full salary on time | 0.352*** | 0.379*** | -0.037 | | 0.57 | | | 1505 | | |  |
|  | (4.11) | (4.65) | (0.38) | |  | | |  | | |  |
| Standard errors clustered at LGA level | | | | | | | | | | |  |
| t statistics in parentheses |  |  |  | | |  | | |  | | |
| * p<0.1, ** p<0.05, *** p<0.01 |  |  | |  | | |  | | |  |  |

Table 8: Impact on Structural Quality of Care, Recordkeeping

|  | PBF  vs  Control | DFF  vs  Control | PBF  vs  DFF | Baseline Mean | N |  |
| --- | --- | --- | --- | --- | --- | --- |
| Proportion of facilities with an up-to-date routine immunization register | 0.083 | 0.132 | -0.050** | 0.90 | 1525 |  |
|  | (1.01) | (1.60) | (2.23) |  |  |  |
| Proportion of facilities with an up-to-date ANC and delivery register | 0.118 | 0.126 | -0.008 | 0.82 | 1489 |  |
|  | (1.30) | 1.38 | (0.17) |  |  |  |
| Proportion of facilities with completed HMIS monthly report | 0.12 | 0.242** | -0.125* | 0.61 | 1525 |  |
|  | (1.28) | (2.57) | (1.90) |  |  |  |
| Standard errors clustered at LGA level |  |  |  |  |  |  |
| t statistics in parentheses |  |  |  |  |  | |
| * p<0.1, ** p<0.05, *** p<0.01 |  |  |  |  |  | |

Table 9: Impact on Structural Quality of Care, Sanitation

|  | PBF  vs  Control | DFF  vs  Control | PBF  vs  DFF | Baseline Mean | | N | |  |
| --- | --- | --- | --- | --- | --- | --- | --- | --- |
| Proportion of health facilities with water for hand washing, soap and clean towel in patient examination area | 0.383*** | 0.484*** | -0.08 | 0.69 | | 1525 | |  |
|  | (4.97) | (6.08) | (1.50) |  | |  | |  |
| Proportion of health facilities with at least one clean and functioning latrine | 0.245** | 0.352*** | -0.064 | 0.60 | | 1525 | |  |
|  | (2.45) | (4.11) | (0.88) |  | |  | |  |
| Proportion of facilities that have a working waste disposal system (bin, pit or incinerator) in use and safety box for sharps | 0.336*** | 0.354*** | -0.074 | 0.61 | | 1525 | |  |
|  | (4.29) | (4.87) | (1.20) |  | |  | |  |
| Standard errors clustered at LGA level | | | | | | | |  |
| t statistics in parentheses |  |  |  | |  | |  | |
| * p<0.1, ** p<0.05, *** p<0.01 |  |  |  | |  | |  | |

Table 10: Impact on Structural Quality of Care, Tuberculosis

|  | PBF  vs  Control | DFF  vs  Control | PBF  vs  DFF | Baseline Mean | N |
| --- | --- | --- | --- | --- | --- |
| Proportion of facilities that offer TB smear test on the day of the survey | 0.003 | -0.028 | 0.017 | 0.17 | 948 |
|  | (0.05) | (0.34) | (0.27) |  |  |
| Proportion of facilities that offer TB diagnosis | 0.191** | 0.177** | 0.04 | 0.16 | 1523 |
|  | (2.30) | (2.27) | (0.63) |  |  |
| Proportion of facilities that offer TB treatment | 0.076 | 0.077 | -0.007 | 0.29 | 1523 |
|  | (1.20) | (1.17) | (0.12) |  |  |
| Standard errors clustered at LGA level | | | |  |  |
| t statistics in parentheses |  |  |  |  |  |
| * p<0.1, ** p<0.05, *** p<0.01 |  |  |  |  |  |

Table 11: Impact on Procedural Quality of Care

|  | PBF  vs  Control | DFF  vs  Control | PBF  vs  DFF | Baseline Mean | N |
| --- | --- | --- | --- | --- | --- |
| Average health worker clinical knowledge score | 0.024 | 0.053 | -0.04 | 0.46 | 1506 |
|  | (0.56) | (1.27) | (1.22) |  |  |
| Under-five examination quality score (based on IMCI protocols) | 0.113** | 0.175*** | -0.076 | 0.57 | 1374 |
|  | (2.16) | (3.24) | (1.34) |  |  |
| ANC examination quality score (based on national ANC protocols) | 0.054 | 0.108** | -0.045 | 0.45 | 1285 |
|  | (1.16) | (2.24) | (1.05) |  |  |
| Proportion of facilities with working means of communication (radio, mobile phone, landline) | 0.662*** | 0.493*** | 0.133* | 0.14 | 1526 |
|  | (5.53) | (4.07) | (1.71) |  |  |
| Proportion of facilities with a working vehicle to transport patients for referral | 0.198*** | 0.208*** | -0.019 | 0.14 | 1526 |
|  | (3.61) | (3.20) | (0.27) |  |  |
| Proportion of health facilities that conduct outreach for key MCH services | 0.252** | 0.190** | 0.072 | 0.45 | 1524 |
|  | (2.65) | (2.06) | (0.75) |  |  |
| Standard errors clustered at LGA level | | | | | |
| t statistics in parentheses |  |  |  |  |  |
| * p<0.1, ** p<0.05, *** p<0.01 |  |  |  |  |  |

Table 12: Impact on Outcomes of Quality of Care, Interactions at Health Facilities

|  | PBF  vs  Control | DFF  vs  Control | PBF  vs  DFF | Baseline Mean | N |
| --- | --- | --- | --- | --- | --- |
| ANC: Average client satisfaction score | 0.03 | 0.058* | -0.035 | 0.89 | 1374 |
|  | (0.99) | (1.98) | 1.25 |  |  |
| U5: Average client satisfaction score | 0.077* | 0.078** | -0.017 | 0.88 | 1284 |
|  | (1.99) | (2.16) | 0.55 |  |  |
| ANC: Proportion of clients who report that facility opening hours are convenient | 0.078* | 0.145*** | -0.071** | 0.91 | 1374 |
|  | (1.78) | (3.17) | 2.1 |  |  |
| U5: Proportion of clients who report that facility opening hours are convenient | 0.070* | 0.115*** | -0.052 | 0.90 | 1284 |
|  | (1.75) | (2.93) | 1.34 |  |  |
| Standard errors clustered at LGA level | | | | | |
| t statistics in parentheses | | | | | |
| * p<0.1, ** p<0.05, *** p<0.01 |  |  |  |  |  |
